# Screening for untreated atrial fibrillation in the elderly population: A community-based study

**DOI:** 10.1371/journal.pone.0269506

**Published:** 2022-06-06

**Authors:** Keitaro Senoo, Arito Yukawa, Takashi Ohkura, Keisuke Shoji, Masao Takigami, Hibiki Iwakoshi, Tetsuro Nishimura, Mitsuko Nakata, Satoshi Teramukai, Satoaki Matoba

**Affiliations:** 1 Department of Cardiac Arrhythmia Research and Innovation, Graduate School of Medical Science, Kyoto Prefectural University of Medicine, Kyoto, Japan; 2 Department of Cardiovascular Medicine, Graduate School of Medical Science, Kyoto Prefectural University of Medicine, Kyoto, Japan; 3 Department of Biostatistics, Graduate School of Medical Science, Kyoto Prefectural University of Medicine, Kyoto, Japan; University of Bologna, ITALY

## Abstract

**Background:**

Strokes are common in people with atrial fibrillation (AF), and can have devastating consequences, especially in the elderly and if AF is untreated. However, community-based studies on screening for untreated AF have not been conducted in Japan, and there has been no evaluation of the effectiveness of early screening for AF in the elderly (≥65 years).

**Methods:**

The Kyoto Prefectural University of Medicine (KPUM) Education Initiative has conducted an AF awareness campaign consisted of screening tests using a blood pressure (BP) monitor with electrocardiogram (ECG) (the Complete, Omron Healthcare Co., Ltd., Kyoto, Japan) and educational lectures for the elderly (≥65 years) from 2019 to 2020. A modeled effectiveness analysis was performed comparing the life-years and QALYs (quality-adjusted life-years) between direct-acting oral anticoagulation (DOAC)-treated AF and untreated AF in a Japanese setting. The basic description of the Markov model was used for the analysis.

**Results:**

A total of 1648 participants were screened, and after excluding those with missing information or data (n = 41), 1607 were finally enrolled. The mean (± standard deviation) age of participants was 72.4±5.8 years, 827 (51.5%) were female, 628 (39.1%) had hypertension, and 1368 (85.1%) had CHA2DS2-VASc score ≥2. After cardiologists’ evaluation of all ECG recordings of the Complete, 15 (0.93%) AF were newly detected. For each AF treated with DOAC, 0.859 QALYs gained over the lifetime for 65 years-old men, and 0.856 QALYs for 65 years-old women compared to non-treatment.

**Conclusion:**

A moderate number of untreated AF were identified in the community-based study. Identification of an increased number of patients with AF, if properly treated with DOAC, ultimately leads to a reduction in the number of strokes occurred over subjects’ lifetime.

## Introduction

Atrial fibrillation (AF) is often asymptomatic or paroxysmal, so it goes undetected and untreated in many patients, putting them at high risk of stroke [1]. AF-related strokes are believed to be more severe and often fatal compared to strokes caused by other factors [2]. AF screening is a major public health issue because early and appropriate anticoagulant therapy could prevent approximately 64% of AF-related strokes [3]. To date, several studies in Western countries have examined opportunistic or systematic AF screening, with AF detection rates of 1–1.4% at a single time point [4] and the usefulness of the screening process for initiating anticoagulation for stroke prevention in newly diagnosed AF patients was proven [5]. Thus, the European Society of Cardiology (ESC) recommended AF screening by palpation or electrocardiogram (ECG) rhythm strip in elderly individuals ≥ 65 years of age [6]. The Japanese guidelines have also been revised in 2020, recommending regular pulse examination and ECG screening for people aged ≥ 65 years of age [7]. However, the detection rate of untreated AF at a single time point in Japanese elderly people aged ≥ 65 years of age is unknown because systematic screening for AF has not been performed in Japan.

The Kyoto Prefectural University of Medicine (KPUM) Education Initiative have been conducting several AF awareness campaigns for people aged ≥65 years from 2019 to 2020. The aims of the initiative were to investigate how much untreated AF patients would be detected if systematic AF screening among the elderly were implemented, and how much QALYs (quality-adjusted life-years) would be affected if AF were subsequently treated by oral anticoagulation (OAC).

## Methods

### Study population

**Fig 1:**
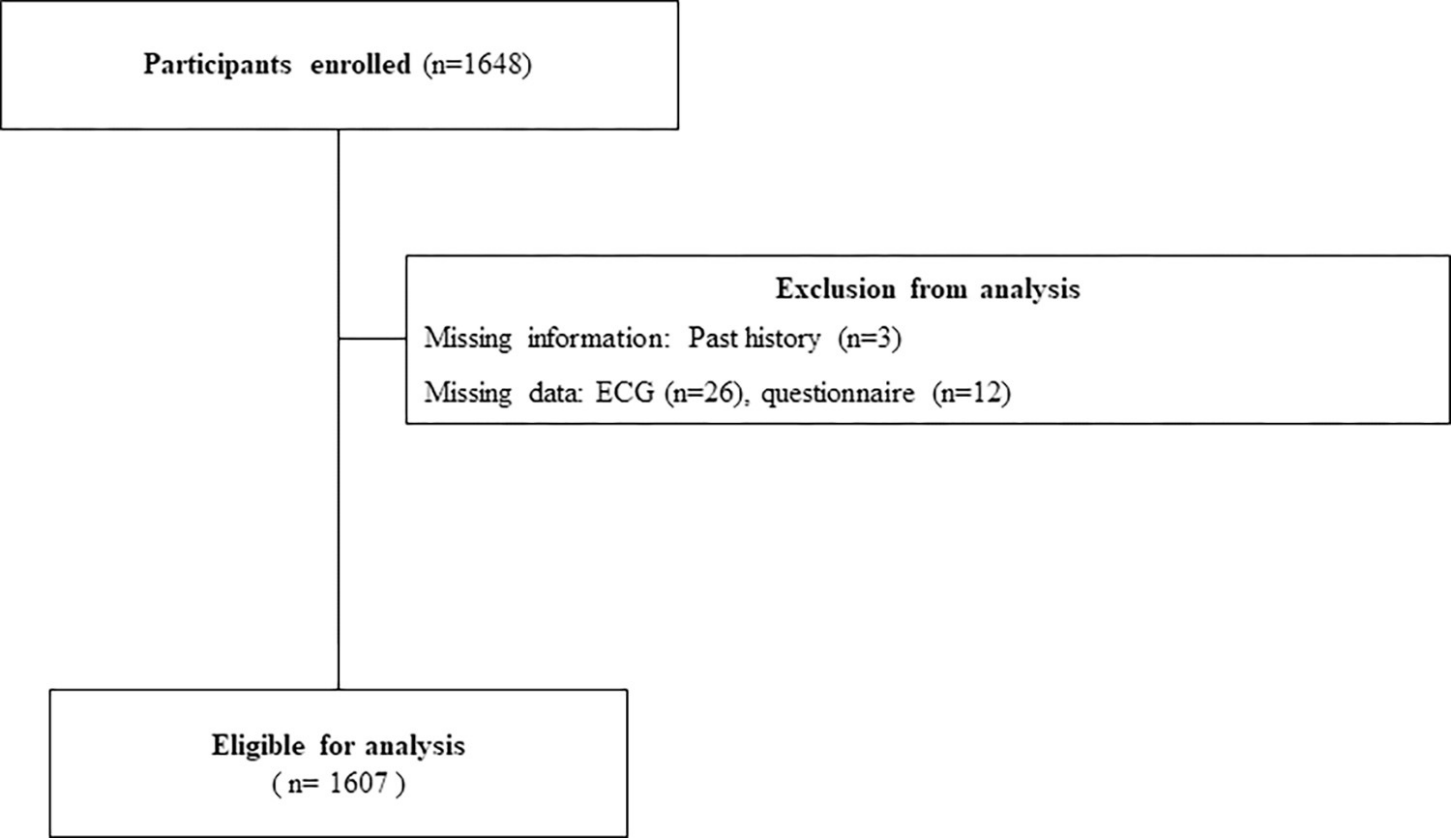
Flow chart of the study.

This study included participants of total 10 AF awareness campaigns for older adults (≥65 years) held between July 2019 and December 2020. Before every symposium, newspaper flyers were mailed to residents in the Kyoto area of Japan to recruit them to the symposium. Residents who mailed letters to the Kyoto Prefectural University of Medicine (KPUM) AF education initiative requesting participation in the symposium were later sent letters describing the research protocol and a consent form. The consent forms were collected on the day of the symposium. A total of 1648 participants were initially screened; after excluding those with missing information (n = 3) and missing data (n = 38), 1607 were ultimately enrolled (**Fig 1**). The present study was approved by the institutional Clinical Research Review Board of Kyoto Prefectural University of Medicine (approval no.: ERB-C-1430) and was conducted in accordance with the Declaration of Helsinki.

### Data collection

Only those who have given the Informed consent were enrolled in the study. Participants first completed a questionnaire on the CHA2DS2-VASc (i.e., congestive heart failure, hypertension, age ≥ 75 years, diabetes mellitus, stroke or transient ischemic attack [TIA], vascular disease, age 65 to 74 years, sex category) before the start of the campaign. Second, they were instructed to perform BP measurement and a 30s ECG recording by using a BP monitor equipped with an attached ECG (Complete, Omron Healthcare Corporation, Kyoto, Japan) [8]. In brief, during BP measurements, a 30s ECG recording was obtained by touching the electrodes located on the top face and both sides of the monitor. Although the Complete has been Food and Drug Administration (FDA)-approved and clinically, this device has demonstrated a sensitivity and specificity of 99% and 86%, respectively, for detecting AF during BP measurements [8], it has not been available in Japan during the study. Therefore, a Cardiologist consultation booth was set up at each AF awareness campaign, and two cardiologists, who were members of the KPUM Education Initiative, read the ECG recordings blindly and classified the rhythm as AF, non AF, or “uninterpretable” (due to baseline artifact, wander, or drift). Kappa (κ) coefficient of interobserver agreement was 0.853. In case of disagreement, a third cardiologist decided the diagnosis afterward. If necessary, patients were advised to contact their general practitioner for further evaluation. Finally, the participants listened to a 30 min educational lecture on AF and stroke by a member of the KPUM Education Initiative.

### Estimation of difference in QALYs between treated AF and untreated AF

**Fig 2:**
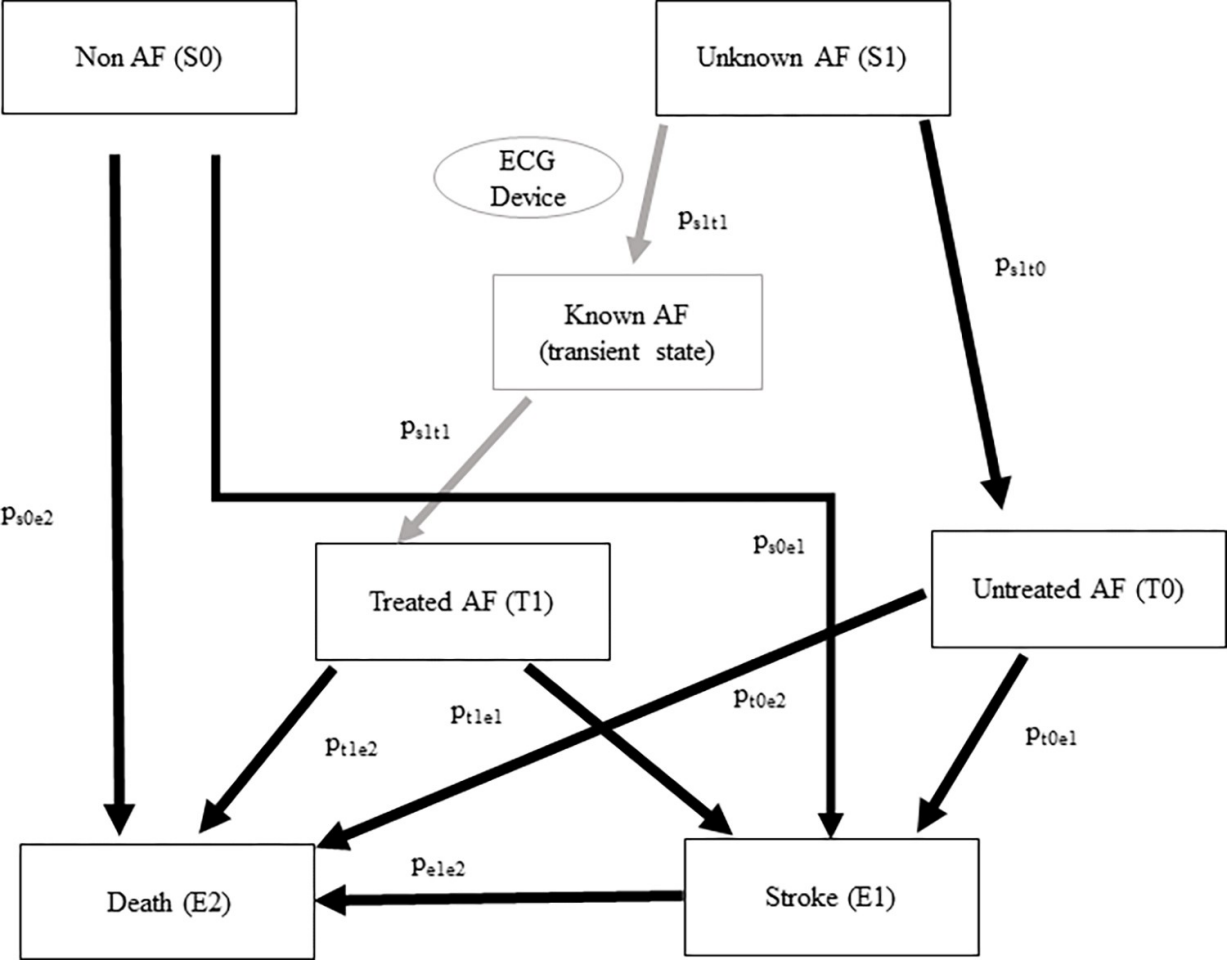
Markov model used in analysis.

A modeled effectiveness analysis was performed comparing the life-years and QALYs between DOAC (direct oral anticoagulant)-treated AF and untreated AF in a Japanese setting. The model assumes following a cohort of men and women aged from 65 to 80 years over a lifetime with annual incident stroke events and death from any cause. The basic description of the Markov model used for the analysis is shown in **Fig 2**. The benefits of DOAC treatment are based on data obtained from ANAFIE [9, 10], GARFIELD-AF registry [11], SHINKEN [12, 13], J-RHYTHM [12], and Fushimi AF [12] registries. Stroke incidence by age and sex was calculated by calibrating the ANAFIE stroke incidence [10] by the stroke incidence in other databases [12, 14]. The mortality for the non-AF population was calculated from Vital Statistics in 2019 [15]. The all-cause mortality for DOAC-treated AF and untreated AF were estimated by multiplying the non-AF mortality by the relative risk obtained from Shinken database for untreated AF [13] and ANAFIE [10] for DOAC-treated AF. The mortality after stroke was obtained from GARFIELD-AF registry [11] and the Japan Stroke Data Bank [16]. The quality of life parameters after stroke were obtained from the Japan Stroke Data Bank [16] and other scientific literature search [17]. The main parameters used to build the analysis were reported in **Tables 1** and **2**. The discount rate was set to 2% according to Central Social Insurance Medical Council guideline [18].

**Table 1:** Estimated annual transition probability between each state.

		nAF->stroke	nAF->death	tAF->stroke*	tAF->death	utAF->stroke*	utAF->death	stroke->death
	Age	p_s0e1	p_s0e2	p_t1e1	p_t1e2	p_t0e1	p_t0e2	p_e1e2
Men	65–69	0.429%	1.318%	1.006%	1.916%	1.381%	2.254%	2.471%
	70–74	0.429%	1.977%	1.006%	2.874%	1.381%	3.381%	3.707%
	75–79	0.715%	3.259%	2.074%	4.738%	2.849%	5.574%	6.112%
	80–84	0.715%	5.865%	2.074%	8.525%	2.849%	10.029%	10.997%
	85–89	1.062%	10.654%	2.655%	15.485%	3.646%	18.218%	19.976%
	90–94	1.062%	18.384%	2.655%	26.721%	3.646%	31.436%	34.469%
	95–99	1.062%	29.693%	2.655%	43.159%	3.646%	50.775%	55.675%
	100-	0.000%	100.000%	0.000%	100.000%	0.000%	100.000%	100.000%
Women	65–69	0.244%	0.543%	1.016%	0.790%	1.395%	0.929%	1.019%
	70–74	0.244%	0.818%	1.016%	1.189%	1.395%	1.399%	1.534%
	75–79	0.551%	1.471%	2.095%	2.138%	2.877%	2.515%	2.758%
	80–84	0.551%	3.008%	2.095%	4.372%	2.877%	5.144%	5.640%
	85–89	1.073%	6.158%	2.682%	8.951%	3.683%	10.530%	11.547%
	90–94	1.073%	12.302%	2.682%	17.880%	3.683%	21.036%	23.066%
	95–99	1.073%	23.173%	2.682%	33.682%	3.683%	39.626%	43.450%
	100-	0.000%	100.000%	0.000%	100.000%	0.000%	100.000%	100.000%
	Reference	14)	15)	9) 10) 12)	10)	9) 10) 12)	13)	11)

nAF: non-AF, tAF: treated AF, utAF: untreated AF

*5.7% in aged 65–74 and 10.1% for aged 75- were estimated to die during the acute stage of stroke

**Table 2:** Estimated QOL parameters.

Decrease in QOL during the acute stage of stroke		Reference
For three months	-0.14	17)
QOL after the acute stage of stroke		
65–74 years of age	0.68	16)17)
75- years of age	0.56	

QOL; quality of life

### Estimation of the cumulative number of preventable strokes in the Japanese population

The cumulative number of strokes (including those who die during the follow-up period) that would be preventable by 10 years of DOAC treatment for AF detected by a hypothetical single screening in all Japanese aged 65–80 years was estimated based on the transition probabilities used for QALY estimation and the observed prevalence of AF cases with a CHAD2 score of 1 or higher. Since the population in Vital Statistics 2019 [15] is classified into 5-year increments, the population for each age group is divided by 5.

### Statistical analysis

Data are expressed as mean ± standard deviation or count and percentage. Comparison of nominal scale data was performed using the Fisher test of independence. Kappa (κ) coefficients for interobserver agreement were calculated, and κ coefficients >0.8 were considered to indicate excellent agreement. Calculations were performed using R software (version 3.6.1; July 5, 2019) and SAS software (version 9.4 the SAS Institute Inc., Cary, NC, USA); differences with P ≤ 0.05 were statistically significant in all analyses.

## Results

### Clinical characteristics of the study population

The mean (± SD) age of the subjects 72.4 ± 5.8 years, 827 (51.5%) were female, 628 (39.1%) had hypertension, and 1368 (85.1%) had a CHA_2_DS_2_-VASc score ≥ 2. Other risk factors for stroke, such as congestive heart failure, diabetes mellitus, previous stroke or TIA and previous vascular disease are summarized in **Table 3**.

**Table 3:** Clinical characteristics of the participants.

Variables	Participants (n = 1607)
Age, mean (SD)	72.4 (5.8)
Gender, female, n (%)	827 (51.5)
Congestive heart failure	34 (2.1)
Hypertension	628 (39.1)
Diabetes mellitus	177 (11.0)
Previous stroke or TIA	43 (2.7)
Previous vascular disease	111 (6.9)
CHADS_2_ score, mean (SD)	0.90 (0.93)
CHADS_2_ score ≥ 2	344 (21.4)
CHA_2_DS_2_-VASc score, mean (SD)	2.5 (1.1)
CHA_2_DS_2_-VASc score ≥ 2	1368 (85.1)

SD; standard deviation, TIA; transient ischemic attack, CHADS2 score; congestive heart failure, hypertension, age ≥ 75 years, diabetes mellitus, stroke or TIA, CHA2DS2-VASc score; congestive heart failure, hypertension, age ≥ 75 years, diabetes mellitus, stroke or TIA, vascular disease, age 65 to 74 years, sex category

### The detection rate of untreated AF

After ECG evaluation by cardiologists, 1547 (96.3%) participants had non AF (sinus rhythm and/or premature atrial contraction [PAC] or premature ventricular contraction [PVC]), 58 (3.6%) had AF and 2 (0.1%) were uninterpretable. After excluding participants with a history of AF (n = 43), 15 (0.93%) participants had untreated AF. The breakdown of CHADS2 score of the 15 untreated AF patients was as follows: 3 patients scored 0, 8 patients scored 1, 2 patients scored 2, 2 patients scored 3, and no patient scored 4 or more. The breakdown of CHA2DS2-VASc score is as follows: 0 patients scored 0, 1 patient scored 1, 5 patients scored 2, 6 patients scored 3, 2 patients scored 4, 1 patient scored 5, and no patient scored 6 or more. The mean CHADS2 and CHA2DS2-VASc scores were 1.2±0.9 and 2.8±1.0, respectively. The number needed to screen (NNS) for one untreated case of AF was 107 (1607/15). The detection rate of untreated AF was 3 times higher in participants with a history of hypertension than those without (1.59% vs 0.51%, respectively; P = 0.035), but did not differ when compared to those with and without other components in the CHADS2 score (**Table 4**).

**Table 4:** Detection rate of untreated AF by each component of CHADS2 score.

Variables		Untreated AF, n		Detection rate, %	P value
		(+)	(-)		
Congestive heart failure, n	+	1	33	2.94	0.28
	-	14	1559	0.89	
Hypertension, n	+	10	618	1.59	0.035
	-	5	972	0.51	
Age≥75 years, n	+	5	519	0.95	1
	-	10	1073	0.92	
Diabetes mellitus, n	+	1	176	0.57	0.90
	-	14	1416	0.98	
Previous stroke or TIA, n	+	0	43	0	1
	-	15	1549	0.96	

AF; atrial fibrillation, TIA; transient ischemic attack

### The estimated difference in life-years and QALYs

The estimated difference in life-years and QALYs are shown in **Table 5**. For each AF treated with DOAC, 0.859 QALYs gained over the lifetime for 65 years-old men, and 0.856 QALYs for 65 years-old women compared to non-treatment. The QALYs gained decreased as age progressed, and 0.710 QALYs gained for 75years-old men and 0.763 QALYs for 75 years-old women. Sensitivity analysis was conducted, changing the discount rate from 0% to 4%, resulting in almost identical results.

**Table 5:** Estimated difference in life-years and QALYs by sex and age.

	Age	Life-Years			QALYs		
		DOAC	No Treatment	Difference	DOAC	No Treatment	Difference
Men	65	13.585	12.858	0.727	13.076	12.217	0.859
	70	11.086	10.405	0.681	10.657	9.867	0.790
	75	8.620	8.001	0.619	8.268	7.558	0.710
	80	6.400	5.858	0.542	6.187	5.593	0.594
Women	65	16.818	16.187	0.631	15.999	15.142	0.856
	70	14.022	13.396	0.626	13.323	12.507	0.816
	75	11.137	10.529	0.607	10.566	9.803	0.763
	80	8.409	7.845	0.564	8.052	7.394	0.658

Discount rate: 2%, QALYs; quality-adjusted life-years, DOAC; direct oral anticoagulant

## Discussion

Several important findings were obtained in this study. For the first time, untreated AF was found in 0.93% of screened subjects aged≥65 years in Japan, most with high stroke risk, and most guideline-eligible for consideration of OACs. The mean CHA2DS2-VASc score of subjects with untreated AF was 2.8, and most had a score of >1, indicating that an individual screened in this way and identified with untreated AF is a likely candidate for OACs due to high stroke risk. Screening for untreated AF increased threefold in hypertensive patients compared with non-hypertensive patients. Identification of an increased number of patients with AF, if properly treated with OAC, ultimately leads to a reduction in the number of strokes occurred over subjects’ lifetime. Finally, the implementation of such a screening program results in about 1 year gain in quality of life in subjects aged ≥ 65 years old men and women.

Opportunistic or systematic AF screening may increase early diagnosis of AF, facilitate an integrated approach that includes appropriate anticoagulation, risk factor modification, and treatment of underlying cardiovascular disease, and reduce complications. Pulse palpation and/or short-term ECG at single time point yielded an AF prevalence of 1–1.4% among the elderly (≥65years) with previously untreated AF, suggesting a NNS of 70 [4]. The present study found that the prevalence of untreated AF was 0.93% in individuals aged ≥65 years, which were comparable to AF screening rates and NNS in Western countries. When the target population was limited to hypertensives aged≥65 years, the detection rate of untreated AF increased significantly to 1.58% compared to non-hypertensives (0.51%), and the NNS drop further to 62.8 (628/10). Importantly, those with untreated AF had a recommendation for anticoagulation based on their stroke risk scores (i.e., CHADS2 scores≥1), according to the current guidelines [6, 7]. Therefore, our data suggest that they can also benefit from AF screening in terms of stroke prevention. Our study examines the effectiveness of screening for AF in the community and shows that it is the feasible option for identifying untreated AF, which is the cause of at least 11.6% of all strokes [1]. As suggested in this study, a higher detection rate of AF would improve the net clinical benefit of increasing the use of OACs, and thus the effect of DOAC use on stroke prevention would be considerably greater [19]. Of course, patient preferences for stroke prevention and tolerance of bleeding risk are important factors to consider in determining how many people decide to take OACs [20, 21]. This paper supports the concept that the use of systematic screening methods can significantly increase the diagnosis of AF and reduce the incidence of stroke. If the findings from our study were extrapolated to the current population of Japan aged 65 to 80 (approx. 26 million people), this would translate to identification of untreated AF in 190,956 people, and the prevention of 7,920 strokes, assuming that all the identified cases were treated with appropriate DOAC (S1 Table).

The lack of patient knowledge is reflected in our studies, where approximately 90% of all participants in this study did not know that AF strokes were more severe than strokes from other causes, and about 70–80% did not know that asymptomatic AF may exist, that even paroxysmal AF is associated with a high risk of stroke, or that anticoagulation is important to reduce the risk of AF strokes. (S2 Table) Understanding the risks and consequences of atrial fibrillation is paramount to adherence to appropriate pharmacotherapy and treatment, and this apparent lack of knowledge is concerning because it calls attention to areas in need of future intervention. Detecting untreated atrial fibrillation through screening is the first step in stroke prevention. However, the lack of knowledge and awareness of AF was also highlighted in this study. To achieve effective stroke prevention, these gaps related to AF need to be addressed and included in policy statements on stroke prevention.

### Study limitations

The results of this study need to be interpreted considering several limitations. Since automated ECG interpretation algorithm in the Complete was not used for physician diagnosis in this study, we did not report on the accuracy of the Complete, which was not the subject of this study. However, to measure the quality of the Complete recordings, we compared the interpretation provided by cardiologists with the Complete automated algorithm (S3 Table). Second, the analysis is based on a Japanese scenario of voluntary subjects attending the screening initiative, who, as volunteers, may have taken advantage of a free screening procedure; hence, the external validity and generalizability of the results presented need to be considered. Third, the transition probabilities from stroke to death are based on those obtained from the GARFIELD study, but the patients who participated in the GARFIELD study may differ from the purely Japanese population. However, the HAS guide for economic evaluation states that it is acceptable to use foreign data for relative risk when the appropriate domestic data is not available [22]. Since GARFIELD data is used for the relative risk of death in AF patients with stroke compared to stroke-free AF patients, the use of foreign data is plausible. Fourth, there are opportunities for early detection of AF outside of screening programs, for example, through ECGs that coincidentally are performed in conjunction with other tests (e.g., preoperative tests for surgical procedures) or monitored ECGs during hospitalization. In particular, older adults are more likely to visit the hospital and undergo inpatient treatment more frequently than younger adults, and thus have more opportunities for incidental detection of AF on ECGs. However, the transition probability model in the present paper is an estimate based on a single systematic screening and does not take into account the incidental detection of AF outside the screening program. Last, the main purpose of this study was to educate older adults about AF and screen for AF, so no cost-effectiveness analysis was performed because it was not designed to address potential costs. However, the modeling analysis of this study suggests that consumer-led AF screening is effective in preventing stroke in the elderly population and increases QALYs. The results in Table 4 also suggest that elderly patients with hypertension may be the optimal target population for AF screening. Therefore, we plan to conduct a large-scale AF screening trial in elderly patients with hypertension to find the optimal population for AF screening based on the cost-effectiveness of stroke prevention in this population.

## Conclusion

In the community-based study, a moderate number of untreated AF cases were identified. Identification of an increased number of patients with AF, if properly treated with OAC, ultimately leads to a reduction in the number of strokes occurred over subjects’ lifetime.

To achieve effective stroke prevention, not only AF screening but also awareness gaps about AF need to be addressed, and research is needed to investigate feasible and cost-effective ways to fill these gaps.

## Supporting information

S1 Table Estimated number of preventable strokes in 10 years among Japanese. Note: b = a x 12 / 1607 (i.e. observed prevalence of CHAD2> = 1), d = 1 / c, e = b / d.
(DOCX)

S2 Table The “9 Key Facts about Atrial Fibrillation” questionnaire. AF; atrial fibrillation, ECG; electrocardiogram.
(DOCX)

S3 Table The quality of the Complete recordings. Thirty-three recordings were labeled as “unclassified” by the Complete algorithm and 2 recordings were uninterpretable by the Complete algorithm. Of the remaining 1572 recordings, the sensitivity and specificity for detecting possible AF by Complete automated algorithm interpretation were 97% (95% CI 0.89–0.99) and 96% (95% CI 0.96–0.96), respectively”.
(DOCX)
