# Supplementary material for: Screening for untreated atrial fibrillation in the elderly population: A community-based study
Source: PLoS One. 2022 Jun 6;17(6):e0269506. doi: 10.1371/journal.pone.0269506 (PMC9170107; doi:10.1371/journal.pone.0269506)
Supplement: S1 Table — Note: b = a x 12 / 1607 (i.e. observed prevalence of CHAD2> = 1), d = 1 / c, e = b / d. (DOCX) [file pone.0269506.s001.docx]

S1 Table. Estimated number of preventable strokes in 10 years among Japanese

| Age at screening | Japanese population in 2019^a^ | | Estimated screen-detected treatable AF^b^ | | Difference in 10years-stroke incidence between DOAC and non-treatment^c^ | | NNT to prevent one stroke^d^ | | Estimated number of preventable strokes in 10 years^e^ | |
| --- | --- | --- | --- | --- | --- | --- | --- | --- | --- | --- |
|  | Men | Women | Men | Women | Men | Women | Men | Women | Men | Women |
| 65 | 838,318 | 892,425 | 6,260 | 6,664 | 3.1% | 3.4% | 33 | 29 | 192 | 230 |
| 66 | 838,318 | 892,425 | 6,260 | 6,664 | 3.2% | 3.7% | 31 | 27 | 203 | 246 |
| 67 | 838,318 | 892,425 | 6,260 | 6,664 | 3.4% | 3.9% | 29 | 25 | 212 | 262 |
| 68 | 838,318 | 892,425 | 6,260 | 6,664 | 3.5% | 4.1% | 28 | 24 | 220 | 276 |
| 69 | 838,318 | 892,425 | 6,260 | 6,664 | 3.6% | 4.3% | 28 | 23 | 227 | 289 |
| 70 | 815,181 | 913,742 | 6,087 | 6,823 | 3.8% | 4.5% | 26 | 22 | 231 | 310 |
| 71 | 815,181 | 913,742 | 6,087 | 6,823 | 3.9% | 4.7% | 26 | 21 | 239 | 323 |
| 72 | 815,181 | 913,742 | 6,087 | 6,823 | 4.0% | 4.9% | 25 | 20 | 245 | 335 |
| 73 | 815,181 | 913,742 | 6,087 | 6,823 | 4.1% | 5.0% | 24 | 20 | 251 | 344 |
| 74 | 815,181 | 913,742 | 6,087 | 6,823 | 4.2% | 5.2% | 24 | 19 | 255 | 352 |
| 75 | 645,093 | 797,261 | 4,817 | 5,953 | 4.1% | 5.2% | 24 | 19 | 198 | 307 |
| 76 | 645,093 | 797,261 | 4,817 | 5,953 | 4.0% | 5.1% | 25 | 20 | 192 | 303 |
| 77 | 645,093 | 797,261 | 4,817 | 5,953 | 3.8% | 5.0% | 26 | 20 | 183 | 295 |
| 78 | 645,093 | 797,261 | 4,817 | 5,953 | 3.6% | 4.8% | 28 | 21 | 171 | 285 |
| 79 | 645,093 | 797,261 | 4,817 | 5,953 | 3.3% | 4.6% | 31 | 22 | 157 | 272 |
| 80 | 438,231 | 623,846 | 3,272 | 4,658 | 3.2% | 4.5% | 31 | 22 | 104 | 210 |
| Subtotal | 11,931,193 | 13,640,984 | 89,094 | 101,862 |  |  |  |  | 3,281 | 4,639 |
| Total | 25，572，177 | | 190,956 | |  |  |  |  | 7,920 | |

Note: b = a x 12 / 1607 (i.e. observed prevalence of CHAD2>=1), d = 1 / c, e = b / d
