# Supplementary material for: Screening for untreated atrial fibrillation in the elderly population: A community-based study
Source: PLoS One. 2022 Jun 6;17(6):e0269506. doi: 10.1371/journal.pone.0269506 (PMC9170107; doi:10.1371/journal.pone.0269506)
Supplement: S2 Table — AF; atrial fibrillation, ECG; electrocardiogram. (DOCX) [file pone.0269506.s002.docx]

S2 Table. the “9 Key Facts about Atrial Fibrillation” questionnaire

| *Question 1-9* | Yes, I knew | No, I did not know | Total |
| --- | --- | --- | --- |
| AF strokes are much more dangerous than other types of strokes | 170 (10.8%) | 1410 (89.2%) | 1580 |
| The risk of stroke is much higher if you have AF that is not treated | 330 (20.9%) | 1251 (79.1%) | 1581 |
| Most AF strokes are caused by a blood clot in the brain | 585 (37.0%) | 996 (63.0%) | 1581 |
| Physicians think about AF stroke risk when suggesting choices about treatment | 273 (17.4%) | 1298 (82.6%) | 1571 |
| Blood thinners can greatly reduce the risk of AF stroke | 393 (25.1%) | 1173 (74.9%) | 1566 |
| Even those with occasional AF are at risk of AF stroke | 302 (19.2%) | 1269 (80.8%) | 1571 |
| Some patients with AF may not have any noticeable symptoms | 265 (16.9%) | 1306 (83.1%) | 1571 |
| Pulse check or portable ECG are useful for early detection of AF | 309 (19.6%) | 1266 (80.4%) | 1575 |
| Lifestyle modification is also essential for the management of AF | 493 (31.3%) | 1083 (68.7%) | 1576 |

AF; atrial fibrillation, ECG; electrocardiogram
