# Supplementary material for: Screening for untreated atrial fibrillation in the elderly population: A community-based study
Source: PLoS One. 2022 Jun 6;17(6):e0269506. doi: 10.1371/journal.pone.0269506 (PMC9170107; doi:10.1371/journal.pone.0269506)
Supplement: S3 Table — Thirty-three recordings were labeled as “unclassified” by the Complete algorithm and 2 recordings were uninterpretable by the Complete algorithm. Of the remaining 1572 recordings, the sensitivity and specificity for detecting possible AF by Complete automated algorithm interpretation were 97% (95% CI 0.89–0.99) and 96% (95% CI 0.96–0.96), respectively”. (DOCX) [file pone.0269506.s003.docx]

S3 Table. The quality of the Complete recordings

| The quality of the Complete recordings | | Cardiologist-interpreted Complete recordings | | | |
| --- | --- | --- | --- | --- | --- |
|  |  | AF | Normal | Uninterpretable | Total |
| Complete algorithm interpretation | Possible AF | 55 | 62 | 2 | 119 |
|  | Normal | 2 | 1453 | 0 | 1455 |
|  | Unclassified | 1 | 32 | 0 | 33 |
|  | Total | 58 | 1547 | 2 | 1607 |

Thirty-three recordings were labeled as “unclassified” by the Complete algorithm and 2 recordings were uninterpretable by the Complete algorithm. Of the remaining 1572 recordings, the sensitivity and specificity for detecting possible AF by Complete automated algorithm interpretation were 97% (95% CI 0.89-0.99) and 96% (95% CI 0.96–0.96), respectively.”
